# Exogenous Melatonin Application Improves Shade Tolerance and Growth Performance of Soybean Under Maize–Soybean Intercropping Systems

**DOI:** 10.3390/plants14152359

**Published:** 2025-08-01

**Authors:** Dan Jia, Ziqing Meng, Shiqiang Hu, Jamal Nasar, Zeqiang Shao, Xiuzhi Zhang, Bakht Amin, Muhammad Arif, Harun Gitari

**Affiliations:** 1 College of Resource and Environment Engineering, Jilin Institute of Chemical Technology, Jilin 132022, China; jiadan@jlict.edu.cn (D.J.); mengziqing1232025@126.com (Z.M.); hushiqiang1232025@163.com (S.H.); 2 Institute of Rice Industry Technology Research, College of Agriculture, Guizhou University, Guiyang 550025, China; jamalnasar554@gmail.com (J.N.); bakht.amin@gzu.edu.cn (B.A.); 3 Institute of Agricultural Resource and Environment, Jilin Academy of Agricultural Sciences (Northeast Agricultural Research Center of China), Changchun 130033, China; 4 College of Agriculture, Guizhou University, Guiyang 550025, China; arif@gzu.edu.cn; 5 Department of Agricultural Science and Technology, School of Agriculture and Environmental Sciences, Kenyatta University, Nairobi P.O. Box 43844-00100, Kenya; hgitari@gmail.com

**Keywords:** shade tolerance, abiotic stress, photosynthetic activities, intercropping, enzymes, growth, yield

## Abstract

Maize–soybean intercropping is widely practised to improve land use efficiency, but shading from maize often limits soybean growth and productivity. Melatonin, a plant signaling molecule with antioxidant and growth-regulating properties, has shown potential in mitigating various abiotic stresses, including low light. This study investigated the efficacy of applying foliar melatonin (MT) to enhance shade tolerance and yield performance of soybean under intercropping. Four melatonin concentrations (0, 50, 100, and 150 µM) were applied to soybean grown under mono- and intercropping systems. The results showed that intercropping significantly reduced growth, photosynthetic activity, and yield-related traits. However, the MT application, particularly at 100 µM (MT_100_), effectively mitigated these declines. MT_100_ improved plant height (by up to 32%), leaf area (8%), internode length (up to 41%), grain yield (32%), and biomass dry matter (30%) compared to untreated intercropped plants. It also enhanced SPAD chlorophyll values, photosynthetic rate, stomatal conductance, chlorophyll fluorescence parameters such as Photosystem II efficiency (ɸPSII), maximum PSII quantum yield (Fv/Fm), photochemical quenching (qp), electron transport rate (ETR), Rubisco activity, and soluble protein content. These findings suggest that foliar application of melatonin, especially at 100 µM, can improve shade resilience in soybean by enhancing physiological and biochemical performance, offering a practical strategy for optimizing productivity in intercropping systems.

## 1. Introduction

Shade is a major abiotic stress that hinders plant growth and development, especially in high-density planting systems such as greenhouses, agroforestry, and intercropping [1,2]. Reduced sunlight, haze, and air pollution further decrease photosynthetically active radiation [3,4]. In response, shaded plants adjust by lowering photosynthetic activity and modifying agronomic traits to cope with limited light [5,6]. However, shading negatively impacts biomass, stem strength, leaf structure, and yield [7]. In intercropping, taller companion plants, such as maize, significantly reduce light availability for legumes, threatening their growth [8,9].

Melatonin is a multifunctional and ubiquitous molecule naturally present in higher plants [10]. In plants, it plays a vital role in enhancing physiological processes such as seed germination and photosynthesis, as well as reproductive traits like flowering, seed setting rate, and yield [11,12,13]. In addition, melatonin contributes to stress tolerance and modulates key metabolic pathways involving lipids, carbohydrates, and nitrogen [10,14]. Due to its wide-ranging influence on plant development and hormone signaling, melatonin has recently been recognized as a potential master regulator of plant growth [15,16]. One of melatonin’s pivotal functions is its ability to enhance photosynthetic performance [10,17]. It achieves this by regulating the expression of the genes associated with Photosystems I and II, thereby improving the efficiency of light capture and energy conversion [10,17]. Critical aspects of photosynthesis, such as light harvesting, chlorophyll biosynthesis, photosynthetic electron transport, and leaf gas exchange, are highly sensitive to environmental stress. Melatonin helps sustain these processes under stress conditions, thereby maintaining overall plant productivity [18].

Melatonin has been increasingly recognized as a multifunctional molecule that enhances abiotic stress tolerance in crops by modulating photosynthetic processes and protecting the photosynthetic machinery [15,17,19]. Various environmental or abiotic stresses often impair the efficiency of the photosynthetic machinery, particularly Photosystem I (PSI) and Photosystem II (PSII) [1,2,20]. This leads to decreased chlorophyll biosynthesis, compromised photochemical efficiency, and reduced carbon assimilation [21,22]. Exotic melatonin application has been shown to counteract such limitations by enhancing the structural stability and functional performance of both PSI and PSII [13,23]. It promotes the repair and regeneration of the D1 protein of PSII, which is critical for sustaining photochemical activity under stress conditions [10,24].

Furthermore, melatonin enhances the content of chlorophyll a and b by upregulating key genes involved in chlorophyll biosynthesis while simultaneously decreasing chlorophyll degradation through its antioxidant action, which prevents oxidative damage to chloroplasts. [10,11,25]. It possesses antioxidant properties that aid in scavenging reactive oxygen species (ROS) produced during stress, thereby sustaining the integrity of chloroplast membranes and safeguarding light-harvesting complexes [10,17,26]. Consequently, crops treated with melatonin exhibit improved photosynthetic efficiency, higher maximum quantum yield (Fv/Fm), and sustained growth under various abiotic stresses, including shaded or low-light conditions [16,27].

These physiological improvements not only enhance plant survival under various stress environments but also contribute to higher biomass accumulation and crop yield [28,29]. Consequently, melatonin emerges as a promising natural biostimulant for improving stress tolerance in crops by optimizing photosystem performance and maintaining chlorophyll content [11,13,25]. However, the impact of melatonin on these physiological indices of soybean under the shading environment of intercropping created by maize has not been explored yet.

In China, maize–soybean intercropping systems are predominantly practiced to increase crop production and utilize the light, water, nutrients, and land resources. This cropping system is cultivated on approximately 667,000 hectares of cultivable land [21,30,31]. However, soybean lodging due to maize-induced shading remains a challenge, limiting yield potential and mechanization compatibility [2,32,33]. Previously, various agronomic practices, including shade-tolerant cultivars [32,34], plant growth regulators [35,36], and appropriate fertilization such as adequate nitrogen [37], titanium [35], and iron and molybdenum application [2] have been used to enhance the physio-agronomic performance of soybean crop under such maize–soybean intercropping systems. However, the effects of melatonin application on soybean growth under intercropping shade stress remain unclear.

This study was purposed to assess the physiological, morphological, and yield responses of soybean to exogenous melatonin application under maize–soybean intercropping conditions. The main objective was to investigate the ability of foliar application of melatonin in enhancing soybean shade tolerance by improving photosynthetic efficiency, chlorophyll fluorescence parameters, and overall growth and yield traits. We hypothesize that foliar application of melatonin, particularly at an optimal concentration (100 µM), would mitigate the negative effects of maize-induced shading on soybean by enhancing its photosynthetic performance, strengthening structural traits, and improving yield-related parameters. Such an enhancement was expected to result from melatonin’s capacity to protect the photosynthetic apparatus, regulate chlorophyll biosynthesis, and support metabolic stability under shade stress.

## 2. Materials and Methods

The field experiment was conducted at the Jilin Institute of Chemical Technology, College of Resource and Environmental Engineering, Jilin, China (43°48′28.59″ N, 125°24′50.38″ E; 248.5 m ASL) over two consecutive growing seasons (2023 and 2024). The region experiences a temperate continental monsoon climate, characterized by four distinct seasons and semi-arid conditions. The experimental soil was loamy with a pH of 6.5 (soil—water = 1:2.5), and contained relatively low levels of alkaline nitrogen (80 mg kg^−1^), total nitrogen (1.5 g kg^−1^), available phosphorus (15 mg kg^−1^), available potassium (115 mg kg^−1^), and organic matter (22 g kg^−1^).

Soybean (*Glycine max* L. ‘Gui Chun 15’) was cultivated as a monocrop (SM) and under intercropping (SI) with maize (*Zea mays* L. ‘Zhengdan 958’), with or without melatonin (MT) foliar application. Melatonin concentrations of 0 µM (MT_0_), 50 µM (MT_50_), 100 µM (MT_100_), and 150 µM (MT_150_) were applied to soybean foliage in three split doses at the V5 (fifth trifoliate), R1 (bloom initiation), and R3 (pod initiation) growth stages.

The concentration gradient of melatonin (50–150 µM) was selected based on a preliminary experiment conducted in the previous season, along with the findings from our earlier studies [10,17,25], which identified 100 µM as an effective dose for improving soybean growth and physiological performance under moderate shading stress conditions. The selected range was intended to evaluate both suboptimal and potentially excessive doses to validate the optimal application rate under intercropping conditions.

The experiment followed a two-factor factorial design, with melatonin concentration (MT) as the main plot and planting pattern (PP) as the subplot, replicated three times. A relay intercropping system was used, where two rows of soybean were intercropped with two rows of maize on 22.5 m^2^ plots (4.5 m × 5 m). The monocropped plots were 18 m^2^ (4.5 m × 4 m). Basal fertilizers were uniformly applied across all the plots: 100 kg ha^−1^ of nitrogen (as urea, 46% N), 100 kg ha^−1^ of phosphorus (as P_2_O_5_, 46% P), and 50 kg ha^−1^ of potassium (as K_2_O, 60% K).

**Figure 1:**
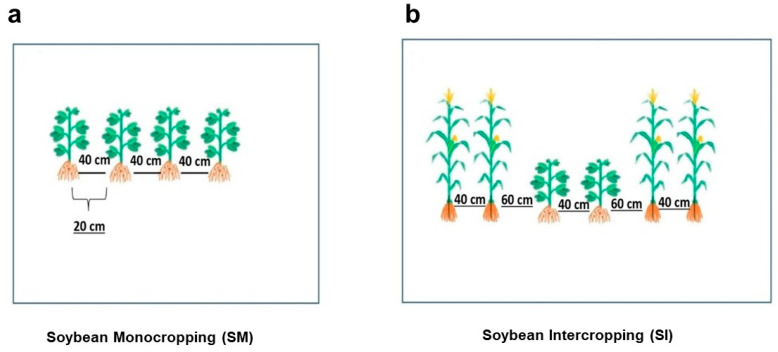
Schematic diagram of the experiment: soybean monocropping (**a**) and soybean intercropping with maize (**b**).

Plant densities were 100,000 soybean plants ha^−1^ and 60,000 maize plants ha^−1^. Row spacing under monocropping was 70 cm for maize and 45 cm for soybean. In the intercropping system, the rows were spaced 60 cm for maize and 40 cm for soybean, with 30 cm between the maize and soybean strips (Figure 1), to reduce interspecific competition.

**Figure 2:**
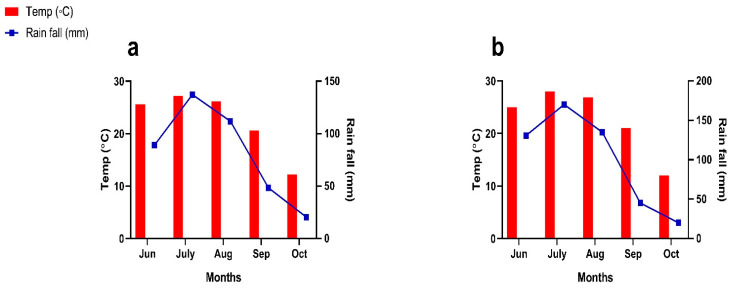
Average temperature and rainfall of the experimental area during the experiment periods of 2023 (**a**) and 2024 (**b**).

The soybean was sown in mid-June and harvested in early October, with the intercropped soybean being planted at the V5 stage of maize development. Standard agronomic practices, including irrigation, pest control, and weeding, were followed throughout. Daily temperature and rainfall data were recorded from sowing to harvesting periods (Figure 2).

### 2.1. Data Collection and Analysis

#### 2.1.1. Growth Indices and Yield Assessment

Soybean growth indices, including stem diameter, stem strength, internode length, plant height, and leaf area, were measured at the V5 (5th leaf) and R5 (seed initiation) stages. Stem strength and internode length were measured using a digital force tester (YYD-1, Zhejiang Top Instrument, Hangzhou, China). Stem diameter was recorded using a Vernier caliper, whereas plant height was measured using a measuring tape. An LI-3000C portable leaf area meter (LI-COR, Lincoln, NE, USA) was used to measure leaf area.

At full maturity, pods were counted per plant, threshed, and weighed to obtain the 1000-grain weight and grain yield, whereas the other straw was sun-dried and then oven-dried for 72 h at 65 °C to determine dry biomass weight.

#### 2.1.2. Chlorophyll SPAD and Photosynthesis

Chlorophyll SPAD values and photosynthetic parameters, including stomatal conductance (gs), photosynthetic rate (Pn), transpiration rate (Tr), and intercellular CO_2_ concentration (Ci), were measured at the V5 (5^th^ leaf) stage [2]. The measurements were taken on fully expanded, healthy leaves between 9:00 and 11:00 a.m. on sunny days using a SPAD-502 Chlorophyll Meter (Minolta, Tokyo, Japan) and a Li-6400XT portable photosynthesis system (Licor Inc., Lincoln, NE, USA). For the photosynthesis measurements, the system light intensity was standardized and maintained at a constant photosynthetically active radiation (PAR) level of 1000 μmol m^−2^ s^−1^ to minimize variability. Leaf temperature was adjusted at approximately 27 °C, and CO_2_ concentration was maintained at 400 μmol mol^−1^ throughout the measurement period [2].

#### 2.1.3. Chlorophyll Fluorescence

Chlorophyll fluorescence parameters, including actual PSII efficiency (ɸPSII), maximum PSII quantum yield (Fv/Fm), photochemical quenching (qp), electron transport rate (ETR), and non-photochemical quenching (NPQ), were measured at night under complete darkness using the Li-6400XT system [2].

#### 2.1.4. Rubisco Activity

At the V5 stage of soybean, Rubisco activity in leaves was analyzed using a Rubisco ELISA kit (Shanghai Fu Life Industry Co., Ltd., Shanghai, China). Frozen leaf samples (1 g) were ground in phosphate buffer with a pH of 7.8, then centrifuged at 7000 rcf for 15 min at 4 °C, and analyzed using a double-antibody sandwich ELISA method. Absorbance was recorded at 450 nm, and Rubisco activity levels were expressed in U/g [38].

#### 2.1.5. Total Soluble Protein

Soluble protein content was determined from 3 g of fresh leaf tissue homogenized in 0.1 M Tris-HCl buffer (pH 8.0) at 4 °C. The extract was centrifuged at 12,000 rpm for 30 min. The supernatant was then used for enzyme extraction. Protein content was assessed using the trichloroacetic acid precipitation method with bovine serum albumin as a standard. Thermostable proteins were further separated via electrophoresis on a 7.5% polyacrylamide gel [39].

#### 2.1.6. Land Equivalent Ratio (LER)

LER was calculated as follows:

$$LER=\frac{Y_{SI}}{Y_{SM}}$$

where Y_SM_ and Y_SI_ epitomize the grain yields of soybean under intercropping and monocropping, respectively. An LER > 1 indicates an intercropping advantage, while LER < 1 suggests competition outweighs benefits [21].

#### 2.1.7. Data Analysis

Data from three replications were processed using MS Excel 2016 and statistically analyzed with Statistix 8.1. A two-way ANOVA was performed to evaluate the effects of melatonin application (MT), planting pattern (PP), and their interaction. Mean comparisons were conducted using the Least Significant Difference (LSD) test at the 5% significance level (*p* ≤ 0.05). Pearson correlation analysis was used to assess relationships among the physio-agronomic indices of soybean. Graphs were created using GraphPad Prism 8.1.

## 3. Results

### 3.1. Growth Indices

Intercropping significantly (*p* ≤ 0.05) suppressed key soybean growth traits, including plant height, leaf area, stem diameter, stem strength, and internode length (Table 1), likely due to shade stress imposed by maize. However, foliar application of melatonin (MT) mitigated these reductions. Notably, MT_100_ treatment led to the most consistent improvements across both years, increasing plant height by 32% (2023) and 23% (2024) compared to untreated intercropped plants (MT_0_). Similarly, MT_100_ enhanced leaf area by 8%, stem diameter by 7% and 6%, and internode length by 35% and 41%, demonstrating its effectiveness in promoting vertical growth under shaded conditions. The observed improvements suggest that MT alleviates shade-induced growth limitations in maize–soybean intercropping systems.

### 3.2. Yield Indices and Biomass Dry Matter

Yield-related traits were also negatively affected by intercropping without MT (*p* ≤ 0.05), reflecting the physiological stress induced by competition for light (Table 2). Nonetheless, the MT application significantly improved the yield and biomass dry matter in intercropped soybean. Specifically, MT_100_ increased grain yield by 25% (2023) and 32% (2024), while biomass dry matter rose by 25% and 30%, respectively, compared to MT_0_. Improvements in 1000-grain weight under MT_100_ were also significant, 24% and 22% higher than untreated plants. These results indicate that MT foliar treatment, especially at 100 µM, can partially offset the yield penalties associated with intercropping stress.

### 3.3. Chlorophyll Content and Photosynthetic Activities

**Figure 3:**
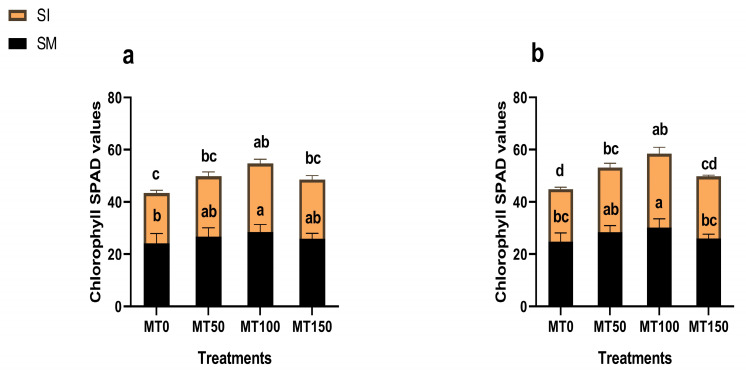
Effect of melatonin (MT) on the chlorophyll SPAD values of monocropping soybean (SM) and intercropping soybean (SI). MT_0_ (0 µM MT), MT_50_ (50 µM MT), MT_100_ (100 µM MT), and MT_150_ (150 µM MT) for 2023 (**a**) and 2024 (**b**). The column bars with dissimilar lowercase letters are significantly different from each other as per the LSD test (*p* ≤ 0.05).

**Figure 4:**
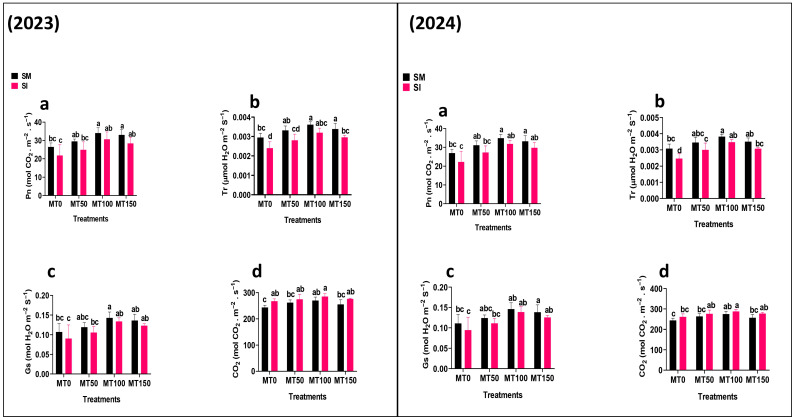
Effect of melatonin (MT) on the photosynthetic activities: Pn: photosynthetic rate (**a**); Tr: transpiration rate (**b**); Gs: stomatal conductance (**c**); and CO_2_: intercellular carbon dioxide (**d**) of monocropping soybean (SM) and intercropping soybean (SI). MT_0_ (0 µM MT), MT_50_ (50 µM MT), MT_100_ (100 µM MT), and MT_150_ (150 µM MT). The column bars with dissimilar lowercase letters are significantly different from each other as per the LSD test (*p* ≤ 0.05).

Photosynthetic pigment levels and gas exchange parameters declined significantly (*p* ≤ 0.05) under intercropping without MT, indicating impaired photosynthetic efficiency due to shading (Figure 3 and Figure 4). However, the MT-treated plants showed marked recovery. MT_100_ notably enhanced the chlorophyll content (SPAD values) by 36% (2023) and 41% (2024) over MT_0_, while increasing net photosynthetic rate (Pn) by 40% and 43%, respectively. Stomatal conductance (gs) and transpiration rate (Tr) also improved significantly under the MT treatments. The observed increase in Ci across the intercropping treatments likely reflects a feedback response to reduced carbon fixation. These improvements in photosynthetic capacity under MT application reinforce its role in enhancing plant performance under light-limited conditions.

### 3.4. Chlorophyll Fluorescence

**Figure 5:**
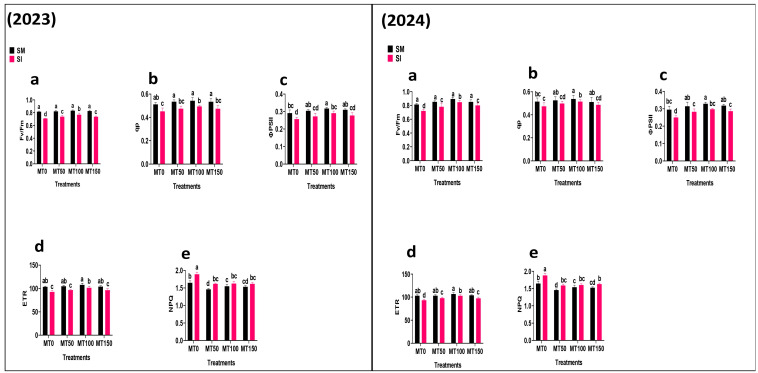
Effect of melatonin (MT) on the chlorophyll fluorescence indicators of monocropping soybean (SM) and intercropping soybean (SI). MT_0_ (0 µM MT), MT_50_ (50 µM MT), MT_100_ (100 µM MT), and MT_150_ (150 µM MT). Fv/Fm: maximum quantum efficiency (**a**); qp: photochemical quenching (**b**); ɸPSII: effective quantum yield of PSII (**c**); ETR: electron transport rate (**d**); NPQ: non-photochemical quenching (**e**). The column bars with dissimilar lowercase letters are significantly different from each other as per the LSD test (*p* ≤ 0.05).

Chlorophyll fluorescence indicators, including Fv/Fm, ɸPSII, qp, and ETR, declined significantly (*p* ≤ 0.05) under intercropping without MT, indicating impaired PSII efficiency (Figure 5). MT application restored these parameters, with MT_100_ showing the strongest effects. For example, Fv/Fm, ϕPSII, qp, and ETR increased by up to 8 and 7%, 9 and 13%, 13 and 19%, and 10%, in 2023 and 2024, respectively, over MT_0_. Conversely, NPQ, which reflects non-photochemical energy dissipation, was highest in untreated intercropped plants, suggesting a stress response. These findings suggest that MT enhances photochemical efficiency and helps maintain energy balance in soybean leaves under intercropping-induced shading.

### 3.5. Rubisco Activity and Soluble Protein

**Figure 6:**
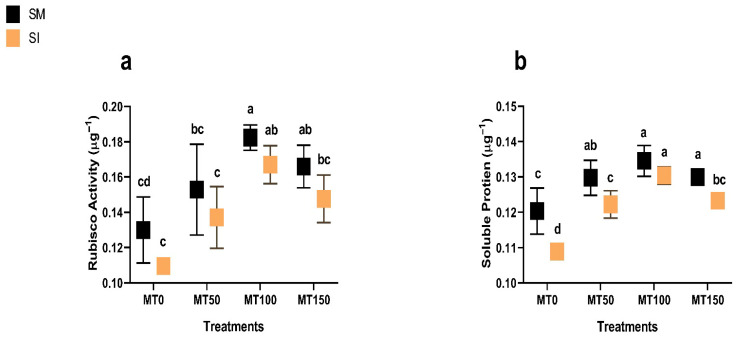
Effect of melatonin (MT) on the Rubisco activity of monocropping soybean (SM) and intercropping soybean (SI) in 2023 (**a**) and 2024 (**b**). MT_0_ (0 µM MT), MT_50_ (50 µM MT), MT_100_ (100 µM MT), and MT_150_ (150 µM MT). The column bars with dissimilar lowercase letters are significantly different from each other as per the LSD test (*p* ≤ 0.05).

**Figure 7:**
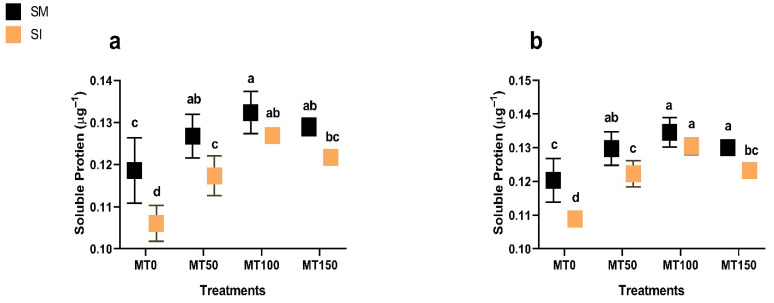
Effect of melatonin (MT) on the soluble protein of monocropping soybean (SM) and intercropping soybean (SI) in 2023 (**a**) and 2024 (**b**). MT_0_ (0 µM MT), MT_50_ (50 µM MT), MT_100_ (100 µM MT), and MT_150_ (150 µM MT). The column bars with dissimilar lowercase letters are significantly different from each other as per the LSD test (*p* ≤ 0.05).

Intercropping reduced both Rubisco activity and soluble protein content significantly (*p* ≤ 0.05), likely due to reduced carbon assimilation under shaded conditions (Figure 6 and Figure 7). However, the MT application substantially mitigated such declines. MT_100_ improved Rubisco activity by over 50% in both years and increased soluble protein content by up to 20%, indicating better preservation of photosynthetic and metabolic function under stress. These results reinforce the conclusion that exogenous melatonin, particularly at 100 µM, supports physiological resilience in soybean under maize–soybean intercropping systems.

### 3.6. Correlation

**Figure 8:**
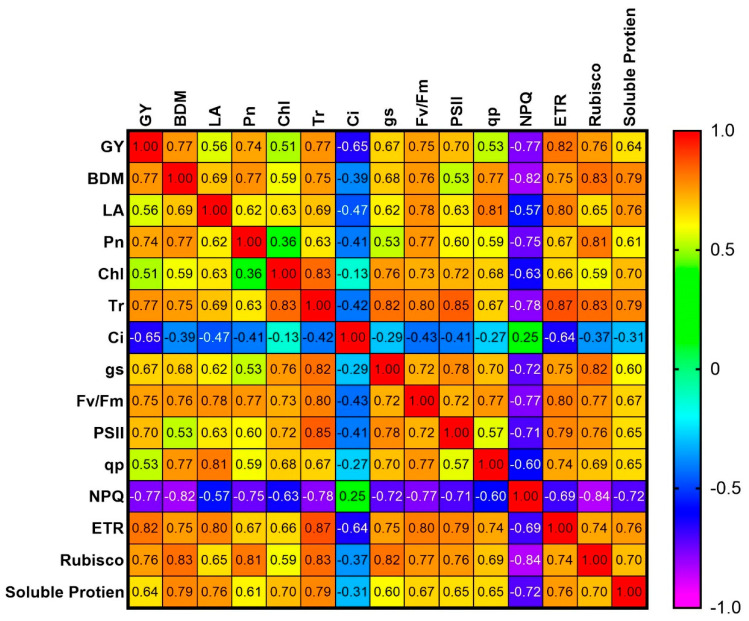
Correlation heat map of physio-agronomic indices of soybean crop under monocropping and intercropping. GY: grain yield; BDM: biomass dry matter; LA: leaf area; Pn: photosynthetic rate; Chl: chlorophyll SPAD values; Tr: transpiration rate; Ci: intercellular carbon dioxide; gs: stomatal conductance; Fv/Fm: maximum quantum efficiency; PSII: effective quantum yield; qp: photochemical quenching; NPQ: non-photochemical quenching; ETR: electron transport rate. Stronger colors and higher positive values indicate strong positive correlations, while lighter colors and negative values reflect weaker or negative correlations among indices.

The correlation analysis revealed that grain yield and biomass dry matter were positively associated with most physiological parameters, including leaf area, chlorophyll content, photosynthetic traits (Pn, gs, and Tr), chlorophyll fluorescence parameters (Fv/Fm, ɸPSII, qp, and ETR), Rubisco activity, and soluble protein content. In contrast, they were negatively correlated with intercellular CO_2_ concentration (Ci) and non-photochemical quenching (NPQ) (Figure 8).

## 4. Discussion

### 4.1. Impact of Melatonin on the Growth and Yield of Soybean

Shade stress is a major abiotic factor in maize–soybean intercropping systems that limits soybean growth by reducing light availability. This shading negatively impacts photosynthetic pigment synthesis, stomatal function, enzyme activity, and biomass accumulation [2,33,35]. The current study confirmed these constraints, as intercropping without melatonin (MT0) significantly reduced plant height, leaf area, stem diameter, and internode length, ultimately lowering yield and biomass. Exogenous melatonin application, particularly at 100 µM (MT_100_), effectively mitigated such negative effects. These improvements likely stem from melatonin’s ability to stimulate cell expansion, maintain chloroplast structure, and regulate hormone levels involved in plant growth [11,14,27]. Notably, the increase in leaf area under MT_100_ facilitated better light capture, contributing to higher chlorophyll content and photosynthetic efficiency. Previous studies have consistently shown that various abiotic stresses, including drought, salinity, cold, and shade, negatively impact the physiological and agronomic performance of plants [16,23,40,41,42]. However, melatonin application has been demonstrated to mitigate such effects and enhance plant growth and yield under stress conditions [11,12,43,44], which is in agreement with our findings.

### 4.2. Effects on Chlorophyll Content and Photosynthetic Activities

Under shaded conditions, chlorophyll biosynthesis is commonly suppressed, leading to reduced SPAD values. Melatonin counteracted this trend, possibly by activating enzymes linked to chlorophyll metabolism and protecting photosynthetic structures from oxidative stress [2,45]. Previously, it has been shown that exogenous melatonin alleviates the negative effects of low light on the chlorophyll content and enhances the activity of Photosystem II in cucumber [46]. Likewise, melatonin significantly improved photosynthetic performance and antioxidant enzyme activities in tomato seedlings exposed to shade [10]. Additionally, melatonin maintains chloroplast integrity and supports gas exchange functions under stress [23]. Consequently, MT-treated plants showed significant improvements in photosynthetic traits, including net photosynthetic rate (Pn), stomatal conductance (gs), and transpiration rate (Tr). The enhancements reflect melatonin’s broader role in maintaining gas exchange and carbon assimilation under suboptimal light, as supported by previous research in drought and salinity contexts [25,47,48].

### 4.3. Impact on the Chlorophyll Fluorescence

The chlorophyll fluorescence measurements further revealed PSII photoinhibition in intercropped soybean without MT. The decline in Fv/Fm, ΦPSII, qp, and ETR, accompanied by increased NPQ, indicates stress-induced inefficiency in energy utilization. MT application reversed these patterns. The increase in fluorescence parameters under MT100 suggests improved PSII stability and reduced excess energy dissipation, possibly due to melatonin’s role in preserving thylakoid membranes and enhancing photoprotective mechanisms [11,16,19]. Such modulation allows more efficient energy conversion and reduced photodamage under shade stress. Similar improvements in PSII function under stress conditions have been reported in maize and soybean, where melatonin treatments sustained higher Fv/Fm and ΦPSII by maintaining D1 protein levels and reducing ROS accumulation [49,50]. In maize, melatonin enhanced photochemical quenching and electron transport rates while decreasing NPQ, indicating more efficient energy use and less photodamage [51]. Additionally, melatonin application was observed to improve PSII efficiency and increase qp and ETR in salt-stressed wheat [52]. These findings highlight melatonin’s regulatory role in photosynthetic apparatus protection and energy conversion efficiency under suboptimal light conditions.

### 4.4. Impact on the Rubisco Activity and Protein Content

Intercropping reduced Rubisco activity and soluble protein content, consistent with impaired photosynthetic machinery under low light. The melatonin-treated plants showed higher Rubisco activity and protein levels, implying enhanced Calvin cycle efficiency and nitrogen metabolism [18,53,54]. Such biochemical improvements may result from melatonin’s capacity to protect enzymes from oxidative degradation, maintain cellular homeostasis, and modulate gene expression related to carbon and nitrogen metabolism [14,16]. These regulatory effects likely contribute to the observed enhancement in physiological performance and yield under intercropping-induced shade stress. These findings are supported by a previous study of Jahan et al. [43], who demonstrated that melatonin enhanced Rubisco carboxylase activity and the expression of carbon assimilation-related genes under drought stress in tomato. Similarly, Zhao et al. [49] observed increased Rubisco and chloroplast protein content in melatonin-treated cucumber under chilling stress. In rice, melatonin upregulated the expression of key Calvin cycle enzymes, including Rubisco activase, under salt stress, thereby sustaining photosynthetic carbon fixation [40]. Moreover, melatonin has been shown to promote total soluble protein synthesis by enhancing the nitrogen uptake and assimilation pathways [55].

### 4.5. Correlation Between Physiological Traits and Yield

The correlation analysis validated these physiological linkages: grain yield and biomass were positively associated with most functional traits—photosynthesis, chlorophyll fluorescence [56], Rubisco activity, and protein content, while negatively associated with stress markers such as intercellular CO_2_ (Ci) and NPQ. This reinforces the fact that melatonin’s beneficial effects are closely tied to its regulation of core photosynthetic and metabolic processes. This study demonstrates melatonin’s effectiveness in reducing shade-induced stress in soybeans, offering a practical tool to enhance performance in intercropping systems.

## 5. Conclusions

This study demonstrates that melatonin application, particularly at 100 µM, alleviates shade-induced physiological and biochemical limitations in soybean under maize–soybean intercropping. Melatonin improved key growth and yield parameters by enhancing the chlorophyll content, photosynthetic gas exchange, chlorophyll fluorescence, Rubisco activity, and protein biosynthesis. Such improvements are underpinned by melatonin’s role in stabilizing photosynthetic systems, regulating energy distribution, and mitigating oxidative damage. Overall, melatonin offers a practical and effective strategy to enhance the shade tolerance and productivity of soybeans in intercropping systems, particularly under low-light environments typical of dense canopies. This suggests that exogenous melatonin, particularly at 100 µM, is a promising strategy to improve soybean productivity under shading stress in maize–soybean intercropping systems.

## Figures and Tables

**Table 1:** Effect of melatonin (MT) on the growth indices of soybean under different planting patterns (PPs).

Year	Treatment		Plant Height (cm)	Leaf Area (cm^2^)	Stem Diameter (mm)	Stem Strength (N)	Internode Length (cm)
	MT	PP					
2023	0	SM	52.40 ± 3.5 ^bc^	251.58 ± 8.5 ^bc^	10.36 ± 0.3 ^bc^	354.84 ± 5.1 ^a^	10.85 ± 1.0 ^c^
		SI	43.34 ± 2.1 ^c^	237.41 ± 8.5 ^d^	9.96 ± 0.4 ^d^	330.10 ± 3.5 ^c^	8.67 ± 1.1 ^d^
	50	SM	60.36 ± 2.5 ^ab^	258.14 ± 7.4 ^ab^	10.70 ± 0.2 ^ab^	358.76 ± 7.0 ^a^	13.27 ± 1.6 ^ab^
		SI	53.33 ± 7.2 ^ab^	248.34 ± 6.2 ^bc^	10.10 ± 0.4 ^c^	336.43 ± 3.5 ^bc^	10.85 ± 1.1 ^c^
	100	SM	61.73 ± 6.6 ^a^	262.77 ± 6.7 ^a^	11.23 ± 0.2 ^a^	361.46 ± 7.0 ^a^	14.13 ± 2.0 ^a^
		SI	57.23 ± 5.7 ^ab^	255.55 ± 4.1 ^b^	10.60 ± 0.5 ^ab^	341.40 ± 4.1 ^b^	12.23 ± 1.0 ^bc^
	150	SM	59.27 ± 7.8 ^ab^	258.91 ± 4.0 ^ab^	10.76 ± 0.3 ^b^	360.70 ± 4.3 ^a^	13.10 ± 1.1 ^b^
		SI	53.77 ± 3.8 ^ab^	244.93 ± 5.0 ^c^	10.26 ± 0.3 ^cd^	334.50 ± 4.0 ^b^	11.63 ± 0.6 ^bc^
	Significance	MT	0.009 **	0.011 *	0.011 *	0.039 *	0.000 ***
		PP	0.008 **	0.001 **	0.000 ***	0.000 ***	0.000 ***
		MT × PP	0.889 ^ns^	0.751 ^ns^	0.904 ^ns^	0.718 ^ns^	0.851 ^ns^
2024	0	SM	57.20 ± 5.9 ^ab^	256.18 ± 9.5 ^b^	10.36 ± 0.7 ^bc^	361.38 ± 3.1 ^b^	10.73 ± 0.7 ^c^
		SI	48.61 ± 3.4 ^c^	238.17 ± 8.3 ^c^	9.96 ± 0.4 ^c^	333.10 ± 2.5 ^e^	8.56 ± 1.1 ^c^
	50	SM	60.50 ± 6.1 ^ab^	259.97 ± 9.1 ^a^	10.70 ± 0.2 ^ab^	363.80 ± 3.5 ^ab^	12.96 ± 1.1 ^ab^
		SI	53.30 ± 2.6 ^bc^	253.34 ± 3.1 ^ab^	10.10 ± 0.3 ^bc^	338.43 ± 3.0 ^d^	10.63 ± 1.4 ^c^
	100	SM	64.86 ± 6.0 ^a^	263.29 ± 8.1 ^a^	11.23 ± 0.2 ^a^	368.01 ± 4.1 ^a^	13.70 ± 1.1 ^a^
		SI	59.96 ± 5.0 ^ab^	257.75 ± 4.0 ^ab^	10.60 ± 0.4 ^ab^	344.86 ± 5.3 ^c^	11.53 ± 1.4 ^b^
	150	SM	58.56 ± 5.2 ^bc^	255.67 ± 5.1 ^ab^	10.76 ± 0.2 ^b^	361.03 ± 2.5 ^b^	12.30 ± 1.1 ^ab^
		SI	54.15 ± 3.3 ^bc^	246.52 ± 6.0 ^bc^	10.26 ± 0.3 ^bc^	340.56 ± 4.1 ^cd^	10.93 ± 1.0 ^c^
	Significance	MT	0.029 *	0.021 *	0.035 *	0.004 **	0.002 **
		PP	0.006 **	0.003 **	0.005 **	0.000 ***	0.000 ***
		MT × PP	0.865 ^ns^	0.426 ^ns^	0.957 ^ns^	0.319 ^ns^	0.872 ^ns^

The means with ± standard deviations (SD) having different lowercase letters (down the column) differ significantly at the LSD test *p* ≤ 0.05 level of probability. MT: melatonin applications; PP: planting pattern; 0: 0 µM melatonin application; 50: 50 µM melatonin application; 100: 100 µM melatonin application; 150: 150 µM melatonin application; SM: Soybean monocropping; SI: soybean intercropping with maize. *** *p* ≤ 0.001; ** *p* ≤ 0.01; * *p* ≤ 0.05; ns: non significance (*p* > 0.05).

**Table 2:** Effect of melatonin (MT) on the yield indices of soybean under different planting patterns (PPs).

Year	Treatment		Grain Yield (kg ha^−1^)	Biomass Dry Matter (kg ha^−1^)	1000-Grain Weight (g)	LER
	MT	PP				
2023	0	SM	1973.74 ± 100.2 ^b^	2893.74 ± 195.3 ^ab^	204.78 ± 15.1 ^ab^	
		SI	1623.00 ± 89.3 ^c^	2180.12 ± 140.0 ^d^	160.94 ± 13.6 ^c^	0.82
	50	SM	2065.97 ± 138.2 ^ab^	2930.23 ± 173.4 ^ab^	210.15 ± 12.6 ^a^	
		SI	1760.12 ± 141.1 ^c^	2366.79 ± 246.0 ^cd^	186.08 ± 22.0 ^b^	0.85
	100	SM	2240.30 ± 105.4 ^a^	3130.13 ± 173.5 ^a^	215.89 ± 16.7 ^a^	
		SI	2033.60 ± 75.0 ^b^	2736.79 ± 248.5 ^b^	199.08 ± 11.6 ^ab^	0.91
	150	SM	2050.33 ± 96.4 ^b^	2661.97 ± 98.0 ^c^	202.89 ± 5.1 ^ab^	
		SI	1749.97 ± 52.2 ^c^	2276.82 ± 165.6 ^d^	183.23 ± 6.2 ^bc^	0.85
	Significance	MT	0.000 ***	0.002 **	0.048 *	
		PP	0.000 ***	0.000 ***	0.000 ***	
		MT × PP	0.681 ^ns^	0.393 ^ns^	0.356 ^ns^	
2024	0	SM	1995.26 ± 90.2 ^c^	3003.60 ± 126.1 ^ab^	212.19 ± 16.5 ^b^	
		SI	1664.18 ± 49.2 ^e^	2273.49 ± 169.1 ^d^	177.93 ± 27.5 ^c^	0.834
	50	SM	2183.89 ± 104.7 ^b^	3180.49 ± 270.2 ^a^	221.60 ± 12.0 ^ab^	
		SI	1873.15 ± 37.3 ^d^	2556.97 ± 220.1 ^cd^	197.29 ± 16.0 ^bc^	0.86
	100	SM	2367.04 ± 84.7 ^a^	3306.78 ± 227.4 ^a^	232.56 ± 14.1 ^a^	
		SI	2200.23 ± 60.8 ^b^	2953.45 ± 144.3 ^ab^	216.82 ± 6.0 ^ab^	0.93
	150	SM	2110.23 ± 20.0 ^bc^	2673.19 ± 258.7 ^c^	221.78 ± 12.2 ^ab^	
		SI	1830.34 ± 62.4 ^d^	2366.82 ± 125.1 ^d^	198.37 ± 12.1 ^bc^	0.86
	Significance	MT	0.000 ***	0.000 ***	0.036 *	
		PP	0.000 ***	0.000 ***	0.001 **	
		MT × PP	0.209 ^ns^	0.425 ^ns^	0.785 ^ns^	

The means with ± standard deviations (SD) having different lowercase letters (down the column) differ significantly at the LSD test *p* ≤ 0.05 level of probability. MT: melatonin application regime; PP: planting pattern; 0: 0 µM melatonin application; 50: 50 µM melatonin application; 100: 100 µM melatonin application; 150: 150 µM melatonin application; SM: soybean monocropping; SI: soybean intercropping with maize. *** *p* ≤ 0.001; ** *p* ≤ 0.01; * *p* ≤ 0.05; ns: non significance (*p* > 0.05).

## Data Availability

The raw data supporting the conclusions of this article will be made available by the authors on request. The data are part of an ongoing project, and their unrestricted release could compromise future publications or experiments. Withholding public access is in accordance with ethical and institutional policies and serves to protect participant confidentiality.
